# Declining Brown-Headed Cowbird (*Molothrus ater*) Populations Are Associated with Landscape-Specific Reductions in Brood Parasitism and Increases in Songbird Productivity

**DOI:** 10.1371/journal.pone.0047591

**Published:** 2012-10-15

**Authors:** W. Andrew Cox, Frank R. Thompson, Brian Root, John Faaborg

**Affiliations:** 1 Department of Fisheries and Wildlife Sciences, University of Missouri, Columbia, Missouri, United States of America; 2 U.S.D.A. Forest Service Northern Research Station, University of Missouri, Columbia, Missouri, United States of America; 3 Missouri Department of Conservation, Columbia, Missouri, United States of America; 4 Division of Biological Sciences, University of Missouri, Columbia, Missouri, United States of America; Hungarian Academy of Sciences, Hungary

## Abstract

Many songbird species have experienced significant population declines, partly because of brood parasitism by the Brown-headed Cowbird (*Molothrus ater*), which is positively associated with increasing landscape forest cover in the midwestern United States. However, cowbirds are also experiencing long-term population declines, which should reduce parasitism pressure and thus increase productivity of host species. We used 20 years of nest monitoring data from five sites in Missouri across a gradient of landscape forest cover to assess temporal trends in the rate and intensity of brood parasitism for Acadian Flycatchers (*Empidonax virescens*), Indigo Buntings (*Passerina cyanea*), and Northern Cardinals (*Cardinalis cardinalis*). We evaluated whether there were concomitant changes in fledging brood size, nest survival, a combination of the two metrics (i.e., host young produced per nest attempt), and whether such changes were more substantial with decreasing landscape forest cover. Parasitism rates and intensities declined substantially during 1991–2010. Fledging brood size and nest survival rates were positively associated with landscape forest cover, confirming the fragmentation hypothesis for Midwest forest birds. Declining parasitism rates were associated with increased fledging brood sizes, with more pronounced increases as landscape forest cover decreased. Nest survival increased insubstantially across time during laying and incubation, but not during the nestling stage. The best predictor of nest survival was parasitism status, with parasitized nests surviving at lower rates than unparasitized nests. Overall, productivity increased during 1991–2010, with more pronounced increases associated with lower levels of landscape forest cover. The negative effects of cowbirds on nest survival in addition to fledging brood size in less forested landscapes suggest that cowbirds may be a primary cause of forest fragmentation effects on songbird productivity in the Midwest. Our results underscore the dynamic nature of demographic parameters, which should be accounted for in predictive models of wildlife responses to future environmental conditions.

## Introduction

Many species of Neotropical migrant songbirds have experienced significant long-term population declines [1], [2]. Identifying causes of the declines is challenging because the life-cycles of migrant songbirds can involve multiple habitat types across vast spatial scales [3]. Nevertheless, conservation biologists have made major advances in our understanding of factors that limit migrant bird populations (reviewed in [4]). On the breeding grounds in eastern North America, habitat loss and fragmentation have decreased songbird productivity by reducing species occurrence and pairing success in small patches [5] and decreasing rates of nest survival [6]. Songbird productivity is furthered hampered by brood parasitism from Brown-headed Cowbirds (*Molothrus ater*; hereafter cowbird), which exhibit increased abundances that result in increased rates of brood parasitism with decreasing regional forest cover in the midwestern United States [7]. In addition to the reduced host productivity occurring as a direct result of parasitism [8], nests with cowbirds may experience greater predation rates because cowbird begging attracts predators [9]. Recent studies of video-monitored nests have also shown that cowbirds are frequent nest predators [10], [11], [12] and that nest predation by cowbirds increases with decreasing regional forest cover [13], which further implicates cowbirds as important drivers of declines in productivity associated with forest fragmentation.

Like many other passerines, cowbirds have exhibited long-term declines in population abundances [14]; the North American Breeding Bird Survey (BBS) indicates a survey-wide 0.6% annual decline (95% CI: −0.9, −0.5%) in cowbird abundance between 1966 and 2010 [15]. Such declines should lead to a reduction in parasitism rates and intensity (i.e., the mean number of cowbird eggs per parasitized nest), both of which are positively correlated with cowbird abundances [16], [17], and potentially even declines in nest predation. Few studies, however, have investigated long-term patterns of parasitism. McLaren et al. [18] reported non-significant declines in parasitism rates in Ontario during 1970–2000 when cowbird abundances in Ontario began to decline, but the authors cautioned that the data they used were not sampled evenly across time or space which limited the strength of their inferences. Rivers et al. [19] documented reduced parasitism rates and intensity on Dickcissel nests (*Spiza americana*) compared to an earlier study at the same location [20], but also noted that the observed patterns may have been a consequence of an increase in the abundance of alternative hosts at their study site.

**Figure 1:**
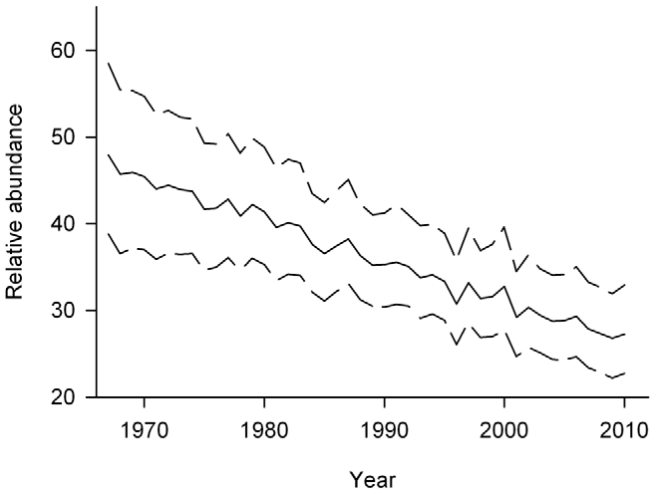
Temporal trend of cowbird abundance in Missouri, 1966–2010. Data are from the North American Breeding Bird Survey [15]. Dashed lines indicate 95% credible intervals.

The decline of cowbirds across time could have substantial positive implications for the conservation of host species. We used a long-term nest monitoring data set to evaluate changes in parasitism rate, parasitism intensity, fledging brood size (i.e., the number of host young per *successful nest*), nest survival rates, and an overall productivity metric that combined fledging brood size and nest survival (i.e., number of host young per *nest attempt*) for three species of forest songbirds across a gradient of forest cover in Missouri, USA, landscapes that have remained largely unchanged during the past 20 years. We predicted that a statewide reduction in cowbird abundances (1.3% annual decline during 1966–2010 [95% CI: −1.9, −0.6%]; Fig. 1;[15]) would result in reduced frequency and intensity of brood parasitism and a concomitant increase in fledging brood size. We further predicted that rates of nest survival would increase across time during the incubation and nestling stages because of reduced nest predation by cowbirds. By contrast, we predicted that nest survival should remain largely unaffected during the laying stage as cowbird abundance changed because a cowbird should lay an egg rather than depredate a nest during that stage and the species we studied rarely abandon an active nest in response to brood parasitism [21, 22, W.A. Cox unpubl. data]. Finally, we predicted that increases in fledging brood size and nest survival should result in increased overall productivity, with more substantial increases occurring in less forested landscapes where cowbirds are most abundant and parasitism rates are highest.

## Methods

### Data Collection

**Figure 2:**
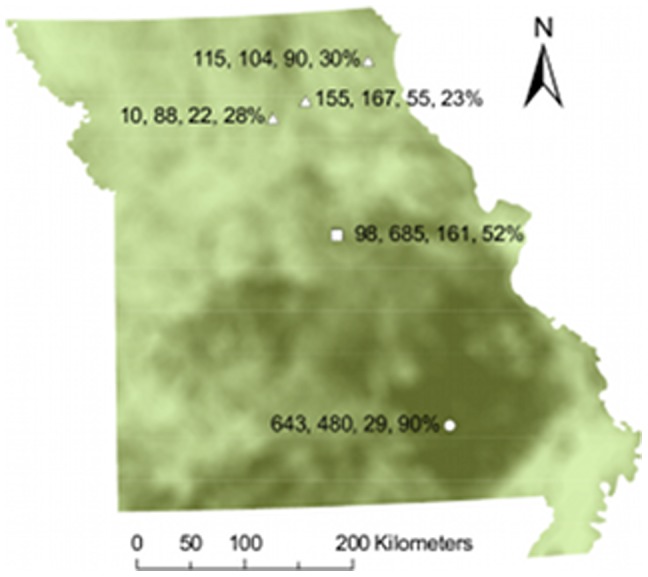
Study locations. Locations of REAM (triangles), Baskett (square), and MOFEP (circle) sites in a study of temporal trends of brood parasitism and nest survival in Missouri, USA, 1991–2009. Shading indicates percent forest cover in a 10-km radius, with darker shading indicating more forest (range: <1–97%). Numbers next to each site indicate number of nests used in nest survival analysis for Acadian Flycatchers, Indigo Buntings, and Northern Cardinals, respectively, and the mean percent forest cover for nests at each site.

We compiled nest monitoring data from multiple studies that occurred on public lands in Missouri, USA, during 1991–2010 (Fig. 2). The Riparian Ecosystem Assessment and Management (REAM) project occurred at three northern Missouri riparian floodplain sites during 1994–2002. The Missouri Ozark Forest Ecosystem Project (MOFEP) is an ongoing study located in the Ozark mountains in southern Missouri where data were collected during 1991–2010 except 1996, in mature secondary successional oak-hickory forest, along roadsides, in even- and uneven-aged timber harvest plots, and in wildlife food plots. Data from the University of Missouri Baskett Research and Education Area (Baskett) were from four separate studies that occurred in old fields and mid- and late-successional oak-hickory forests during 1992–2002 and 2007–2010. All studies were permitted by the Missouri Department of Conservation and the U.S. Fish and Wildlife Service as required by law. We limited our analysis to three songbird species that were well represented at all study sites. The Indigo Bunting (*Passerina cyanea*, hereafter bunting) builds nests in shrubs within old fields, along forest edges, and in dense forest understory vegetation. The Acadian Flycatcher (*Empidonax virescens*, hereafter flycatcher) is a forest-interior species that typically nests in the branches of sub-canopy trees. The Northern Cardinal (*Cardinalis cardinalis*, hereafter cardinal) is a habitat generalist that nests in shrubs and trees at a variety of heights in old fields and wooded habitats. Researchers used systematic search or behavioral cues to find nests [23] and usually monitored them every 1–4 d until the nest fledged or failed. We considered a nest to be parasitized if ≥1 cowbird egg or nestling was in the nest at any point while being monitored. We considered parasitism intensity to be the maximum number of cowbird eggs and/or nestlings observed in a nest on any monitoring visit. To minimize the potential for bias in survival estimates, we right-censored nests with unknown or questionable fates (i.e., >4 d between the penultimate and final nest check and no evidence of fledglings or signs of predation noted) as well as those that failed because of researcher activities [24].

We obtained geographic coordinates recorded from handheld GPS units (*n* = 1,186) or from nest locations marked on gridded maps in a GIS (*n* = 959). One study at Baskett did not have exact nest locations mapped but did record subplot locations (e.g., specific old fields or forest sections); we defined the center of the subplot as the location for these nests (*n* = 718). The remaining nests were assigned locations based on the center of a breeding territory (*n* = 45), or an estimated location based on written descriptions (*n* = 4).

We used landscape forest cover as a metric of habitat fragmentation (*sensu* [6]). We assessed temporal changes in landscape forest cover to determine whether it was appropriate to use a single National Land Cover Database (NLCD) for all of our nests and to determine whether the hypothesized changes in parasitism rates and intensity could be due to broad-scale habitat change (i.e., increased forest cover). First, we downloaded U.S.D.A. Forest Service Forest Inventory Analysis (FIA) data (http://www.fia.fs.fed.us/) for all the counties that fell within a 10-km radius of our nests and assessed forest cover change from 1989 (data were not available for 1990–1991) to 2010. Second, we downloaded two NLCD land cover change databases (http://www.mrlc.gov/; 1992 versus 2001 and 2001 versus 2006) that correct for compatibility issues between releases and identify pixels that have changed between releases. We used the pixels within the 10-km buffers surrounding nests that changed to or from forest to calculate the percent change in forest cover.

We concluded it was appropriate to use the 2001 database to calculate landscape forest cover for all nests to avoid compatibility issues between different NLCD releases (see results). We used ArcMap 9.3 [25] to reclassify land cover as forest (composed of deciduous, evergreen, and mixed forests, shrub/scrub [a category which included transitional forests], and woody wetlands) or non-forest (all other land types). We used the focal statistics tool to calculate percent forest surrounding each pixel and chose a 10-km radius because it best explains variation in nest predation [26] and is a strong predictor of parasitism risk and cowbird abundance [7], [26], [27] for forest songbirds in the United States.

### Analysis

Our overall approach was to evaluate sets of candidate models explaining variation in nest parasitism rates, parasitism intensity, fledging brood size, and nest survival within an information-theoretic approach [28]. We then combined model-based predictions of nest survival and fledging brood size using a bootstrapping approach to estimate the effects of forest cover and year on the number of young produced per nest attempt (hereafter productivity).

We modeled parasitism rates using logistic regression with a binary response variable (parasitized versus unparasitized) with the GLIMMIX procedure in SAS [29]. We constructed five candidate models (Table 1) representing hypotheses explaining variation in parasitism rates and ranked models by calculating Akaike's Information Criterion (AIC) values and the difference between the top model and other candidate models (ΔAIC). We then used the ΔAIC values to calculate weights (*w_i_*) and evaluate the relative support of each model in the candidate set. All models included study site as a random effect to acknowledge the potential for correlated fates within sites and to account for site-specific variation in parasitism rates. All models except the null also included host species and nest site habitat type, both of which can influence parasitism rates [6], [7]. We assigned nest site habitat types by collapsing the original researchers' designations into three categories (forest, clearcut, non-forest). If the original researcher did not designate a habitat type, we used the nest location within a GIS to assign the habitat type. Non-forested habitat included fields, old fields, forest/field edges, and roadsides. The MOFEP experiment also includes group selection and thinning harvest regimes (see [30] for details), but because treatments retained substantial tree canopy and do not influence cowbird abundance [31], we assigned all nests in those stands to the forest habitat type. We included landscape forest cover and year as fixed effects of interest in candidate models. We also included an interaction term (forest cover × year) in one model to assess the hypothesis that the decline in parasitism rates would be more substantial in fragmented landscapes compared to highly forested landscapes. We did not consider an interaction between species and year because all three species exhibit pronounced increases in parasitism rates with decreasing forest cover in Missouri (W.A. Cox, unpubl. data) likely because of increased cowbird abundances across the same gradient. We assessed the same model set for parasitism intensity using the number of cowbird eggs or young as a normally distributed response variable, an approach that is most robust to deviations from an assumed distribution when analyzing egg or nestling count data [32].

**Table 1:** Model selection results from analysis of temporal trends of brood parasitism and productivity (top-ranked models have weights in bold) for Indigo Buntings, Acadian Flycatchers, and Northern Cardinals in Missouri, USA, 1991–2010.

Model structure	Parasitism rates (*n* = 2,912)				Parasitism intensity (*n* = 578)				Productivity (*n* = 952)			
	*K* a	AIC^b^	ÄAIC^c^	*w_i_* d	*K*	AIC	ÄAIC	*w_i_*	*K*	AIC	ÄAIC	*w_i_*
Species + habitat type + forest cover + year	8	2,386.20	0.00	**0.62**	9	1,078.35	0.00	**0.68**	10	2,389.52	0.50	0.31
Species + habitat type + (forest cover × year)	9	2,387.13	0.93	0.38	10	1,080.00	1.65	0.30	11	2,389.98	0.96	0.24
Species + habitat type + forest cover	7	2,409.97	23.77	0.00	8	1,085.79	7.44	0.02	9	2,389.02	0.00	**0.39**
Species + habitat type	6	2,425.68	39.48	0.00	7	1,089.82	11.47	0.00	8	2,392.97	3.95	0.05
Null	2	2,541.66	155.46	0.00	3	1,100.87	22.52	0.00	3	2,651.07	262.05	0.00

a Number of parameters. Parasitism rate models include a parameter for estimating covariance structure of the random effect (study site), whereas intensity and productivity models also include a parameter for the residual variance of the random effect. All productivity models except the null also include a term for parasitism status. Models with interactions include all constitutive terms as per [58].

b Akaike's Information Criterion.

c Difference between the AIC score of current and top-ranked model.

d Relative weight of support for the model.

Because interpretation of model-averaged coefficients is problematic when some parameters occur as both additive and interactive terms in various models [28], and because we needed parasitism rates to produce estimates of nest survival and fledging brood size (see below), we generated model-averaged predictions rather than coefficients from the sets of candidate models. We held terms in our regression models at specified values (e.g., an equal proportion of all three species; see [33] for an overview of empirical versus model-based estimation) to produce predictions that were not biased by our sample, which was uneven across some covariates.

We used nests for which exact counts of host young were known to model fledging brood size as a function of forest cover and year. We used the same approach and model set as described for parasitism rates and intensity (Table 1), but because parasitized nests typically fledge substantially fewer young than unparasitized nests [8], all models except the null also included a term for parasitism status. We covaried values for parasitism rate, forest cover, and year according to the predictions produced from our parasitism rate analysis to produce predictions of fledging brood size.

We used the logistic exposure method [34] within an information-theoretic framework [28] to model nest survival using the NLMIXED procedure in SAS [29]. All models (Table 2) included a random-effect term for study site and all but the null model included fixed-effect terms for the nuisance parameters (songbird species, nest stage, and habitat type) as each influences rates of survival in our study system [35], [36]. We included a term for brood parasitism status in all but a true null model and a model with just the nuisance parameters because parasitized nests may be under greater risk of predation [9]. We included a model with landscape forest cover and year to evaluate whether landscape-specific rates of predation (i.e., low nest survival in fragmented habitats) changed across time, and a term with a year × forest cover interaction to assess our hypothesis that overall predation rates would decline in more fragmented landscapes where cowbirds are more abundant but remain unchanged in highly forested landscapes where cowbirds are less abundant. We included a model with a stage × year interaction to assess the hypothesis that if temporal trends in nest survival were caused by cowbirds, they would occur during incubation or nestling stages because cowbirds should lay eggs rather than depredate nests during the laying stage (the species we studied infrequently abandon nests because of brood parasitism [21, 22, W.A. Cox unpubl. data]). Finally, we included a global model with all constitutive terms from the candidate models.

**Table 2:** Model selection results from analysis of temporal trends of nest survival for Indigo Buntings, Acadian Flycatchers, and Northern Cardinals in Missouri, USA, 1991–2010.

Model structure	*K* a	AIC^b^	ÄAIC^c^	*w_i_* d
Species + stage + habitat type + parasitism status + forest cover	10	10,230.96	0.00	0.24
Species + (stage × year) + habitat type + parasitism status + forest cover	13	10,231.14	0.18	0.22
Species + stage + habitat type + parasitism status	9	10,231.41	0.45	0.19
Species + (stage × year) + habitat type + parasitism status + (forest cover × year)	14	10,231.42	0.46	0.19
Species + stage + habitat type + parasitism status + forest cover + year	11	10,232.80	1.84	0.09
Species + stage + habitat type + parasitism status + (forest cover × year)	12	10,233.15	2.19	0.08
Species + stage + habitat type	8	10,292.25	61.29	0.00
Null	2	10,363.32	132.36	0.00

a Number of parameters. All models include a parameter for estimating covariance structure of the random effect (study site) and models with interactions include all constitutive terms as per [48].

b Akaike's Information Criterion.

c Difference between the AIC score of current and top-ranked model.

d Relative weight of support for the model.

To assess temporal changes in productivity, we combined fledging brood size and nest survival estimates. To do this, we used a parametric bootstrapping approach that allowed us to incorporate the error associated with the fledging brood size and nest survival estimates [37]. First, we generated model-averaged predictions for fledging brood size and model-averaged predictions for nest survival across years. Then for each year, we randomly selected a value for fledging brood size and a value for nest survival probability from a normal distribution of possible values that was constrained by each term's standard error. We then calculated productivity as fledging brood size × nest survival probability. We repeated this 10,000 times and treated the mean value as a point estimate of productivity and the 2.5% and 97.5% values as the confidence limits. All analyses were performed using SAS [29]. Mean values are presented with standard errors unless otherwise indicated.

## Results

**Figure 3:**
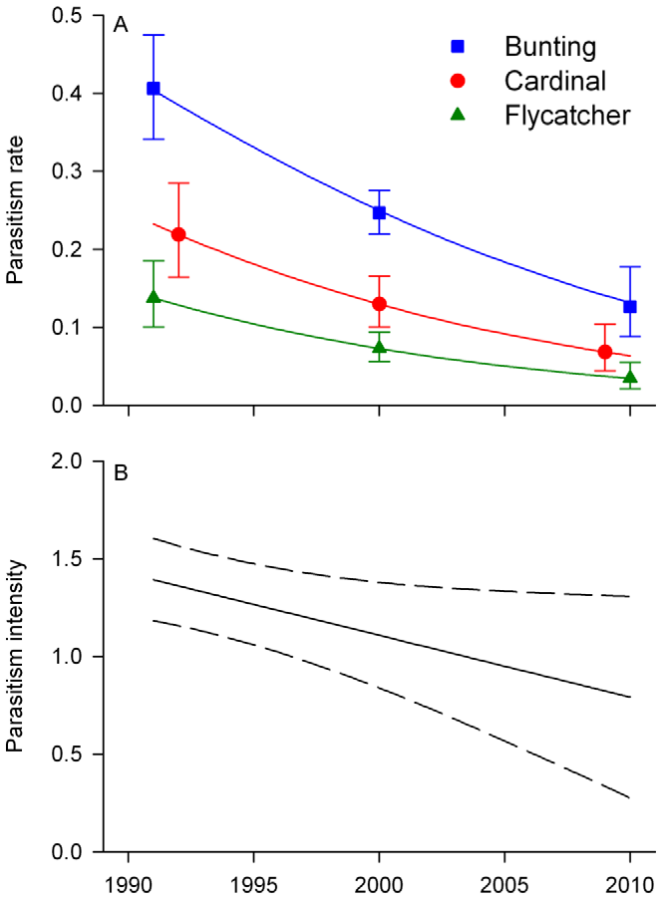
Temporal trends in parasitism rates and intensity. Model-based predictions of Brown-headed Cowbird parasitism rates (A) and intensity (B) for three songbird species in Missouri, USA, 1991–2010. Rates are for a population at the median value for forest cover (53%) and at observed frequencies of nests in three habitat types (forest, clearcut, non-forest). Rates in (B) are for a balanced population of the three songbird species. Intensity values <1 are not possible (i.e., there cannot be <1 cowbird egg or nestling in a parasitized nest), so predictions of parasitism intensity should be interpreted with regard to temporal changes rather than absolute values. Error bars in (A) and dashed lines in (B) represent 95% confidence intervals, with some bars offset for visual clarity.

**Figure 4:**
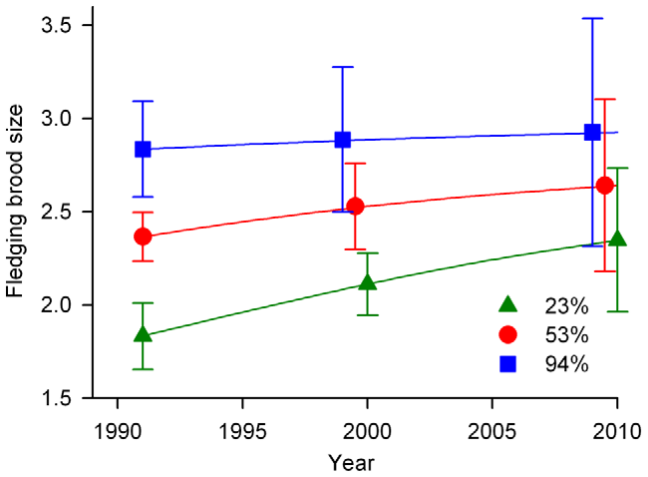
Temporal trends in fledging brood size. Model-based predictions of fledging brood size at 5%, median, and 95% observed values of forest cover (23%, 53%, and 94%, respectively) across a balanced population of three songbird species in Missouri, USA, 1991–2010. Estimates are for a population at observed frequencies of nests in three habitat types (forest, clearcut, non-forest), with parasitism rates varying across years and forest cover levels on the basis of the parasitism rate model-selection analysis. Error bars represent 95% confidence intervals and are offset for visual clarity.

Forest cover in the 10 counties surrounding our nests increased 7% (640,628 ha to 682,725 ha) between 1989 and 2010 according to FIA data. In contrast, NLCD data from a 10-km radius surrounding each nest suggested that forest cover declined by 1.8% from 1992 to 2001, and by 0.4% from 2001 to 2006. Of the nests monitored wherein contents were reliably observed, 423 of 1,524 (28%) bunting nests, 76 of 1,021 (7%) flycatcher nests, and 79 of 367 (22%) cardinal nests were parasitized. All three species were well represented across the gradient of forest cover (Fig. 2; bunting mean: 58±1%, range: 23–95%; flycatcher mean: 70±1%, range: 23–95%; cardinal mean: 44±1%, range: 23–95%). Forest cover strongly influenced parasitism rates with a mean predicted parasitism rate of 33% (95% CI: 28–37%) for a population of nests balanced across species at 23% forest cover (the 5^th^ percentile of observed forest cover values) versus 3% (95% CI: 2–4%) for a population of nests at 94% forest cover (the 95^th^ percentile). A parameter for year was in the two best-supported parasitism rate models, which combined for 100% of the overall weight of evidence (Table 1). Parasitism rates differed between species and declined across time (Fig. 3a). A parameter for year was also in the top two parasitism intensity models, which combined for 98% of the overall weight of evidence (Table 1). The mean number of cowbird eggs per parasitized nest differed among species (buntings: 1.43±0.03; flycatchers: 1.09±0.04; cardinals: 1.23±0.06) and declined across time (Fig. 3b). There was a substantial effect of brood parasitism on fledging brood size; fledging brood sizes were greater for unparasitized versus parasitized nests for buntings (2.79±0.12 versus 1.47±0.14 fledglings), flycatchers (2.50±0.13 versus 1.17±0.15 fledglings), and cardinals (2.82±0.15 versus 1.50±0.16 fledglings). The best-supported model for fledging brood size did not include a covariate for year, but there was considerable model-selection uncertainty (Table 1), and model-based predictions of fledging brood size increased across time (Fig. 4) because the predictions incorporated the negative association between parasitism rates and year. The effect size of the increase in fledging brood size from 1991 to 2010 was greater for nests as forest cover declined, with a 22% increase in brood size for nests at 23% forest cover (1.83±0.09 in 1991 versus 2.35±0.19 in 2010) compared to a 3% increase for nests at 94% forest cover (2.83±0.13 versus 2.93±0.31; Fig. 4).

**Figure 5:**
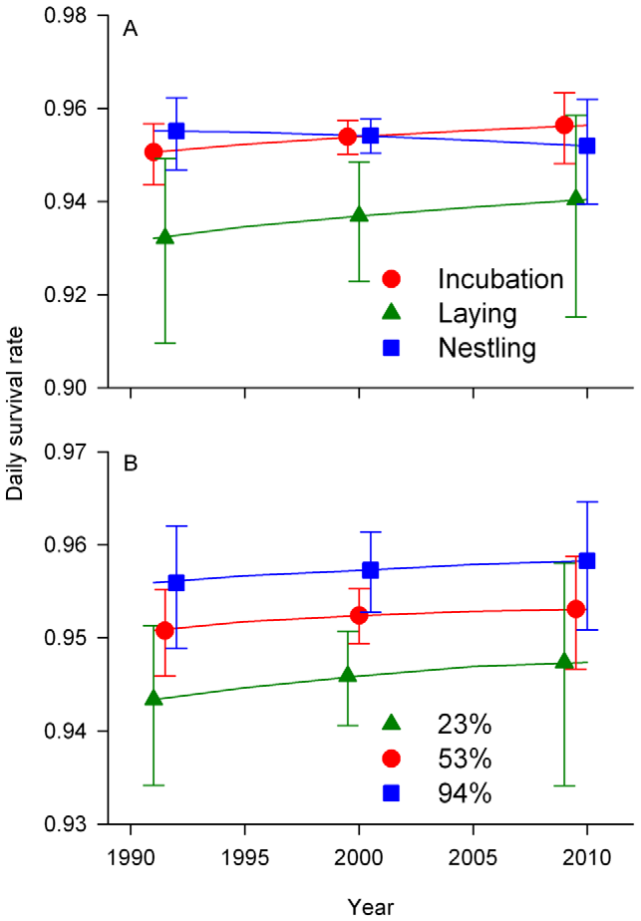
Nest survival by stage and forest cover. Model-based predictions of daily songbird nest survival rate by nest stage (A) and for 5%, median, and 95% observed values of forest cover (23%, 53%, and 94%, respectively; B) in Missouri, USA, 1991–2010. Predictions in (A) are for a balanced population of three songbird species at the median (53%) value of forest cover and at observed frequencies of nests in three habitat types (forest, clearcut, non-forest). Predictions in (B) are for a balanced population of three songbird species and three habitat types, with unbalanced nest stages (lay: 11%, incubation: 49%, nestling: 40%) that reflect the average time spent in each stage across species. Parasitism rates were varied across years and forest cover levels on the basis of the parasitism rate model-selection analysis. Error bars represent 95% confidence intervals and are offset for visual clarity.

The total effective sample size [38] for our nest survival analysis was 33,698. There was considerable model-selection uncertainty, with the best-supported model having 24% of the overall weight of evidence (Table 2). There was limited support for our prediction that temporal variation in nest survival was nest stage-specific. A model with a stage × year interaction term was the second-best supported in the candidate set, but overall models with this term only had 41% of the cumulative AIC weight. Model-averaged estimates of nest survival for each stage were contrary to our predications; there was an insubstantial increase in nest survival across time during laying and incubation stages but not during the nestling stage (Fig. 5a). There was also limited support for our prediction that temporal trends in nest survival would be landscape-specific. The forest cover × year interaction term did not appear in any of the top three models (Table 2), and although there was a small increase in overall nest survival across time, the difference between landscapes was relatively constant (Fig 5b). Instead, the best predictor of nest survival was parasitism status, as evidenced by the substantial improvement in model likelihood between the model with species, stage, and habitat type variables (AIC = 10,292.25) and the same model that also included parasitism status (AIC = 10,231.41; Table 2). This effect was not due solely to nest abandonment or the total loss of host young to cowbirds, as a post hoc analysis that included only successful nests and those that failed because of nest predation indicated that the period survival rate (i.e., cumulative survival probability across all three nest stages) for parasitized nests (0.14, 95% CI: 0.11–0.18) was substantially lower than for unparasitized nests (0.26, 95% CI: 0.24–0.29).

**Figure 6:**
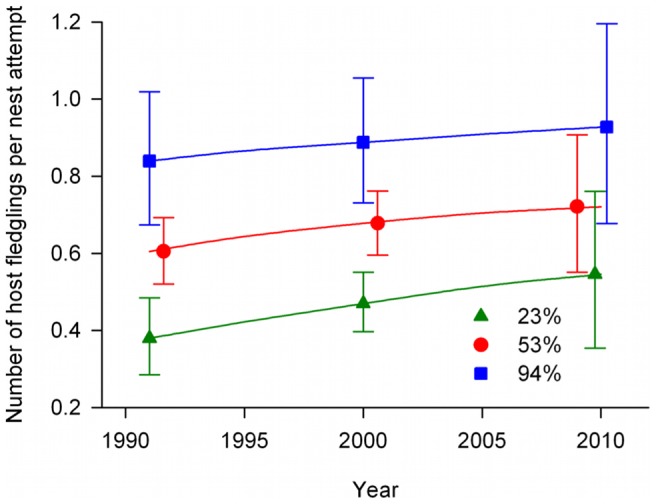
Productivity per nest attempt. Average productivity per nest attempt across time at 5%, median, and 95% observed values of forest cover (23%, 53%, and 94%, respectively) for a balanced population of three songbird species in a study of brood parasitism and nest predation in Missouri, USA, 1991–2010. Error bars represent 95% confidence intervals and are offset for visual clarity.

The influence of landscape forest cover on fledging brood size and nest survival led to a substantial increase in overall productivity as forest cover increased (Fig. 6). Similar to the fledging brood size results upon which estimates were partly based, the effect size of the increase in productivity from 1991 to 2010 was larger as forest cover declined, with a 30% increase in productivity for nests at 23% forest cover (0.38, 95% CI: 0.21–0.48 in 1991 versus 0.55, 95% CI: 0.35–0.76 in 2010) compared to a 10% increase for nests at 94% forest cover (0.84, 95% CI: 0.67–1.02 versus 0.93, 95% CI: 0.68–1.20; Fig. 6).

## Discussion

Several factors may have contributed to cowbird declines in Missouri during 1966–2010. Cowbird abundances increase with proximity to grazing livestock [39], and cattle production in Missouri in 2009 declined >40% from its peak in 1975 [40]. Further, although landscape forest cover remained largely unchanged at the 10-km scale surrounding the nests we monitored, increased forest cover throughout Missouri (FIA data suggest an 11% increase in forested acreage between 1989 and 2010) may also reduce regional cowbird densities by reducing habitat used for foraging and/or increasing the distance between spatially distinct foraging and breeding habitats [41]. Other factors that limit bird populations such as broad climatic patterns [42] may also affect cowbird abundances. Regardless of the mechanisms driving cowbird declines, our data from 20 years of monitoring nests at five Missouri sites suggest productivity of three songbird species increased concurrent with these declines. Our results also provide further evidence of the negative effect of forest fragmentation on songbird productivity, though these effects may be changing over time.

Concordant with documented declines in cowbird abundance, the rate and intensity of parasitism declined substantially during 1991–2010 for the three species we studied. Parasitized nests of most passerine bird species exhibit reduced host productivity [8], an effect that is more pronounced in nests with >1 cowbird nestling [43]. Declines in the rate and intensity of parasitism between 1991 and 2010 at our study sites resulted in increased predicted fledging brood sizes as forest cover declined, where cowbirds are most abundant and parasitism rates are highest. Population dynamics of songbirds are most sensitive to changes in adult and post-fledging survival, but fledging brood size can be an important determinant in population stability [44].

Because parasitism rates increase and nest survival rates decrease with increasing forest fragmentation in the Midwest [6], and because cowbirds are established nest predators that depredate nests more frequently in fragmented landscapes [13], we predicted that nest survival rates would increase as cowbird abundances decreased across time, more so in fragmented landscapes where cowbirds are abundant. Further, we predicted that changes in nest survival would be stage-specific because cowbirds should have little incentive to depredate nests when hosts are laying eggs. However, nest survival only increased modestly over time, and there was considerable uncertainty surrounding our predictions (Fig. 5b). Further, nest survival increased in a manner contrary to both of our predictions. This is perhaps unsurprising given the diverse suite of predators known to contribute to overall rates of predation in our study system [35], [36] that also have exhibited long-term population changes. For example, Blue Jays (*Cyanocitta cristata*) are frequent predators during incubation [35] and have declined 1.1% annually in Missouri during 1991–2010 (95% CI: −2.0, −0.3%; [15]), which may contribute to the temporal patterns of predation we observed during the laying and incubation stages. By contrast, populations of Broad-winged Hawks (*Buteo platypterus*) and Barred Owls (*Strix asio*), frequent predators that depredate nests almost exclusively during the nestling stage [35], may have increased substantially in Missouri during 1991–2010 (Broad-winged Hawk: 4.3% [95% CI: −0.5, 9.2%], Barred Owl: 5.8% [95% CI: 3.3, 8.9%]; [15]). Correlations between broad-scale predator population trends and local demographic metrics should be interpreted with caution, but they are concordant with previous studies relating predator abundance and avian reproductive performance [45].

Lower nest survival and reduced fledging brood sizes associated with low landscape forest cover led to a substantial negative correlation between forest cover and our combined productivity metric (i.e., host young produced per nest attempt). With predicted productivity values ≥50% lower at 23% forest cover compared to 94% forest cover regardless of year (Fig. 6), our data serve as a stark reminder of the negative effects of forest fragmentation on breeding birds in the midwestern United States as described by Robinson et al. [6]. We suggest that cowbirds may be an under-acknowledged mechanism behind reduced nest survival in fragmented landscapes. The presence of a cowbird nestling can result in zero host young fledging from on otherwise successful nest because of egg removal or destruction (e.g., [46]), host ejection [47], or host mortality due to competition [48]. In addition, louder and more frequent begging by cowbird young [9] and increased parental activity at nests with cowbird young [49] may result in higher predation risk [50] and contribute to lower rates of nest survival for parasitized nests as seen here and elsewhere [51], [52]. The negative effects of parasitism on fledging brood size and nest survival (and thus on our productivity measure) combined with the fact that cowbirds depredate nests more frequently in less forested landscapes [13] suggest that cowbirds may be a primary cause of the forest fragmentation effect on songbird productivity in the Midwest.

Despite the substantial decline in predicted parasitism rates during 1991–2010, the concomitant increase in productivity was comparatively modest because the strongly negative impact of parasitism on the productivity of a single nest is muted across a population of nests wherein most are not parasitized and many parasitized nests are depredated. Nevertheless, lower landscape forest cover was associated with a greater increase in predicted productivity, which provides support for our hypothesis that temporal trends in productivity should be landscape-specific. It also suggests that some habitat patches that were formerly population sinks (*sensu* [53]) may now produce enough young to be considered sources, which exemplifies the potential value of incorporating temporally dynamic source-sink models into the management of migratory songbirds.

We stress that the patterns we observed in Missouri almost assuredly do not apply throughout the extensive range of the Brown-headed Cowbird. The BBS data suggest that temporal trends in cowbird abundances are not uniform; 19 U.S. states have seen substantial cowbird declines between 1966–2010, but 12 states have had significant increases in cowbird abundances during the same timespan [15]. Furthermore, current abundance trends may not persist into the future, as it is possible that current cowbird declines are part of a long-term population oscillation. Only seven states had significant declines during 2000–2010, and even though cowbird abundance has declined in Canada throughout the entire BBS survey period (1.5% annual decline [95% CI: −2.2, −1.0%]), they actually increased in abundance during 2000–2010 (2.4% annual increase [95% CI: 0.9, 4.1%]; [15]). Finally, Missouri is near the historical center of the cowbird breeding range [54], [55] where abundances are typically highest [17], and it may be difficult to detect biologically meaningful changes in the effect of cowbirds on productivity in locations where cowbird abundances are substantially lower.

Predictive models designed to assess the effects of climate change on wildlife distributions and abundances into the next century are increasingly common in the literature. Such models will be most useful when they incorporate important biotic interactions [56], [57] such as those between brood parasites and their host species. Given the inherent complexity of virtually all ecosystems, any such model is likely to be surrounded by substantial uncertainty. Nevertheless, our study underscores the fact that critical demographic parameters are not static in time but can instead exhibit long-term temporal trends. Demographers rely upon empirical data collection to parameterize their models, but our data demonstrate that values derived from older studies may not be reflective of current or future conditions. Studies such as this that monitor populations across extended time periods are essential if we are to accurately predict future trends in the abundance, occurrence, or productivity of wildlife.
